# Environmental justice and REDD+ safeguards in Laos: Lessons from an authoritarian political regime

**DOI:** 10.1007/s13280-021-01618-7

**Published:** 2021-09-14

**Authors:** Sabaheta Ramcilovic-Suominen, Sophia Carodenuto, Constance McDermott, Juha Hiedanpää

**Affiliations:** 1 Natural Resources Institute Finland, Itäinen Pitkäkatu 4 A, 20520 Turku, Finland; 2 University of Victoria, Victoria, Canada; 3 University of Oxford, Oxford, UK

**Keywords:** REDD+, Environmental justice, Laos, Social safeguards, Authoritarian regimes

## Abstract

Balancing agendas for climate mitigation and environmental justice continues to be one of the key challenges in climate change governance mechanisms, such as Reducing Emissions from Deforestation and Degradation (REDD+). In this paper we apply the three-dimensional environmental justice framework as a lens to examine the REDD+ process in the Lao People’s Democratic Republic (Laos) and the REDD+ social safeguards. We focus particularly on challenges to justice faced by marginalized communities living in forest frontier areas under an authoritarian regime. Drawing on policy analysis and open-ended interviews across different policy levels, we explore procedural, distributional, and recognitional justice across the REDD+ policy levels in Laos. We find that REDD+ social safeguards have been applied by both donors and state actors in ways that facilitate external control. We underscore how authoritarian regime control over civil society and ethnic minority groups thwarts justice. We also highlight how this political culture and lack of inclusiveness are used by donors and project managers to implement their projects with little political debate. Further obstacles to justice relate to limitations inherent in the REDD+ instrument, including tight schedules for dealing with highly sensitive socio-political issues under social safeguards. These findings echo other research but go further in questioning the adequacy of safeguards to promote justice under a nationally driven REDD+. We highlight the importance of recognition and political context, including aspects such as power relations, self-determination and self-governance of traditional or customary structures, in shaping justice outcomes.

**Supplementary Information:**

The online version contains supplementary material available at 10.1007/s13280-021-01618-7.

## Introduction: REDD+ social safeguards and the importance of justice in REDD+

Reducing Emissions from Deforestation and Degradation (REDD+) is a nationally driven, performance-based climate mitigation and finance mechanism negotiated under the United Nations Framework Convention on Climate Change (UNFCCC). The core logic of REDD+ is to leverage international public and private sector finance to compensate the REDD+ developing countries for reducing deforestation and forest degradation. Funding for REDD+ may come from diverse sources, including multilateral and bilateral aid agencies and international finance institutions, as well as private sector engagement in carbon markets and other market-based activities. REDD+ is ‘performance-based’, meaning that payments are based on results in the form of verified reductions in forest emissions. It is also ‘nationally driven’, meaning that national governments in REDD+ countries have sovereign authority to choose whether to participate in REDD+, determine their own priority REDD+ actions, ensure adherence to various safeguards, and control how REDD+ funds are distributed within their borders. In order to receive results-based funds, however, national governments should first engage in a range of donor-supported ‘readiness’ activities, such as developing national REDD+ strategies and aligning forest sector policies and practices in support of REDD+ objectives. While the content of REDD+ strategies in principle depends on the context-based drivers and agents of deforestation, most of the national REDD+ strategies include land use and forest planning, tenure and ownership reform, development of carbon measuring, reporting and verification (MRV), and measures for benefit sharing across policy actors, including local forest communities (Angelsen 2008, 2009).

The focus of REDD+ on payments for forest carbon (with forests predominantly state-owned) combined with its emphasis on national sovereignty has raised an array of concerns over social and environmental justice. It has been feared, for example, that transforming forest carbon into an internationally traded commodity could drive land grabbing and other forms of dispossession of local and Indigenous Peoples, loss of local livelihoods, and loss of biodiversity (Corbera 2012; McDermott et al. 2012). With its focus on sovereign authority, REDD+ could also drive the recentralization of state control (e.g., Phelps et al. 2010), which could be a matter of particular concern in authoritarian states (e.g., Pham et al 2014; Hoang et al. 2019).

To address such concerns, a set of seven REDD+ social and ecological “safeguards” were established at the Conference of Parties (COP) 16, in Cancun (UNFCCC 2011). The “Cancun safeguards”, also known as “REDD+ safeguards”, outline prerequisites for an environmentally and socially responsible, or ‘just’, REDD+. The following year, the UNFCCC COP 17 in Durban introduced Safeguard Information Systems (SIS) as a mechanism for REDD+ countries to demonstrate compliance with the REDD+ safeguards. At COP 19, the SIS was established as eligibility criteria for result-based payments, meaning that the countries need to prove adherence to the safeguards to qualify for result-based payments. Guidance on how this is done in practice, however, remains unclear, which in turn leads to ad hoc and diverse national approaches to demonstrating adherence to safeguards (Jagger et al. 2014).

Of the seven REDD+ safeguards,^1^ those that most directly speak to social justice are (i) respect for the knowledge and rights of Indigenous Peoples and local communities, (ii) full and effective participation of Indigenous Peoples and local communities, and (iii) enhancement of environmental and social benefits and local livelihoods. These safeguards aim to strengthen participation of non-governmental actors and local communities, improve distribution of burdens and benefits, and enhance cultural and political recognition and thereby ensure socially just outcomes. Yet delivering such outcomes in practice has been extremely challenging. REDD+ efforts, especially in Southeast Asia, have been critiqued for a wide range of shortcomings, including a lack of performance on social, environmental, and climate goals (Sanders et al. 2017; Milne et al. 2019), the continuation of neocolonial policy and practice (Bumpus and Liverman 2011), depoliticization and perpetuation of conflicts (Myers et al. 2018; Milne et al. 2019), and omission of non-carbon and social benefits in the implementation stage (Sanders et al. 2017; Ramcilovic-Suominen and Nathan 2020). Such challenges are particularly acute in forest frontiers, often inhabited by indigenous groups, migrants, and/or ethnic minorities (Smith and Dressler 2019; Brockhaus et al. this issue), and in authoritarian states concerned with expanding state control (e.g., Brown and MacLellan 2020; Bruun 2020).

In response, an increasing body of literature has focused explicitly on the justice and equity dynamics of REDD+ (e.g., Nathan and Pasgaard 2017; Suiseeya 2017; Dawson et al. 2018; Satyal et al. 2018). For example, Hoang et al. (2019) and Nathan and Pasgaard (2017) examine how Vietnamese and Cambodian authorities have selectively employed the international ‘politics’ of climate justice to de-legitimize and curtail swidden agriculture in upland frontiers rather than address large-scale commercial agriculture as a driver of forest loss in lowland areas. In addition, there are observations on how REDD+ processes in Ethiopia, Nepal, and Vietnam have been used in ways that centralize state control over forest resources (Hoang et al. 2019; Brown and MacLellan 2020; Satyal et al. 2018). In partial response to these critiques, various authors have argued that REDD+ has also created new opportunities for norm contestation, which could have positive effects on justice in the longer term (Suiseeya 2017), even in authoritarian states (Pham et al. 2014). Yet to highlight the possibility that REDD+, in particular contexts and points in time, might promote justice for some actors, risks obscuring its dominant negative effects on vulnerable actors, and the lack of effectiveness of REDD+ safeguards in protecting or empowering vulnerable actors.

Consequently, there is a scientific demand for detailed but holistic empirical assessments of justice to understand whether REDD+ safeguards are either enhancing or undermining justice and for whom. This paper provides such analysis by applying a three-dimensional environmental justice framework to elucidate how REDD+ in authoritarian settings affects marginalized groups. While scientific knowledge on REDD+ in Laos is accumulating, existing studies frequently address singular aspects of justice^2^ and hence fail to provide a holistic understanding of how the different dimensions of justice relate to and influence one another. We adopt a framework that intertwines justice across three dimensions: procedural (participation and representation of different actors), distributional (distribution of burdens and opportunities), and recognitional (recognition of cultural diversity and power asymmetries and rights, including the right to self-organize). We focus on the heightened challenges to justice faced by marginalized communities living in forest frontier areas under an authoritarian regime. Our work contributes to theoretical debates on environmental justice by highlighting how recognition as well as politics, such as power asymmetries and cultural and political self-determination, affect procedural and distributive justice. Focusing on political aspects and recognition clarifies the risk that REDD+ safeguards, as introduced by international actors and implemented through ‘nationally driven’ processes, may not only fail to achieve local justice, but may further endanger actors on the political margins of the state.

## Politics of exclusion and REDD+ in Laos

As a post-socialist, one-party state formed in 1975, Laos has a history of weak political participation, limited democratic representation, and freedom of speech (Evans 2002; Stuart-Fox 2005, 2007; Creak and Barney 2018). The lack of robust regulation, transparent governance of natural resources (Fujita and Phengsopha 2012; Singh 2012), and respect for human rights (Amnesty International 2016; Gindroz 2017; IFHR 2017) are often used to describe the institutional and political context in the country. The strict authoritarian style of governing is the legacy of the Lao People’s Revolutionary Party (LPRP, or the Party) which with the Political Bureau (Polit Bureau) in principle rules the country (Stuart-Fox 2006, 2007; Creak and Barney 2018). The Polit Bureau and the Party influence and control political life and the major economic sectors, including forestry and agriculture (Stuart-Fox 2007; Creak and Barney 2018). In the international arena of REDD+,  the country is represented by the Government of Laos (GoL) which, however, is tightly linked to the Party (Croissant and Lorenz 2018).

Public participation and the right of citizens to organize in civil society organizations (CSOs) is restricted. The state controls civil society, promulgating laws that limit their participation and freedom to act. The conditions have significantly worsened with Degree on Associations No. 238, enacted in 2017, which makes registration and independent operation of domestic CSOs nearly impossible (GoL 2017; IFHR 2017). This latest law prescribes the areas with which CSOs can and cannot engage and imposes stringent monitoring of their activities and finances, which are monitored through annual reporting to the Ministry of Home Affairs that can consequently discontinue CSO registration and right to operate (No. 238/PMO 2017). Domestic and international activists working on land and forestry issues continue to risk imprisonment and abduction (Amnesty International 2016; Gindroz 2017; Sims 2017). Public participation is mainly orchestrated by government-sponsored and established ‘mass organizations’. These are organs of the state operating at national, provincial, district, and village level, which are considered to be directly accountable to the state (Stuart-Fox 2006) and commonly engaged in forest projects in the villages, including REDD+ projects.

At the local level, the government uses less coercive measures, including promises of development and progress in the villages, but also local rituals as a means to consolidate authority and control (Singh 2014). Limiting shifting cultivation is framed as progress and as a matter of development. The ‘ritual governance’ is a welcoming soft strategy, but it has been criticized for celebrating only the cultural heritage of the ethnic majority groups that identify as ‘Lao Loum’, or lowland dwelling people (Singh 2014). A critical and contextual reality is the historically rooted cultural stigma, discrimination, and political marginalization of ethnic minorities, such as the Hmong and Akha. This discrimination and marginalization relates to their practice of shifting cultivation and opium production (Baird and Shoemaker 2007) and, in the case of Hmong people, to their alliance with the USA and against the Lao People's Revolutionary Party (LPRP) in the Lao civil war (early 1960s to 1975)^3^ (Baird and Shoemaker 2007; Singh 2012; Ramcilovic-Suominen 2019).

To monitor and control the ethnic minorities and their cultivation practices, the government of Laos operates a vast resettlement program that aims to relocate the ethnic minorities from the uplands to the lowlands and closer to roads, markets and infrastructure (Ducourtieux et al. 2005; Baird and Shoemaker 2007; Baird et al. 2009). The resettlement program is also used as a nation-building strategy that often takes place at the expense of cultural assimilation of ethnic minorities (Baird and Shoemaker 2007). Given that the government of Laos does not recognize the ethnic minority groups as Indigenous, the reference to UNDRIP in the REDD+ social safeguards and FPIC process, which focuses explicitly on Indigenous People, is of little assistance to Lao ethnic minority groups.

Concerning shifting cultivation, it is important to highlight that the practice, as well as its purposes, varies enormously. Many people practice shifting cultivation in addition to other land uses and do so for both subsistence and commercial purposes (Cramb et al. 2009; Fox et al. 2009, 2014). The continuing state and donor-imposed limitations on shifting cultivation in Laos and the rest of Southeast Asia are well documented elsewhere (see Lestrelin et al. 2012; Kenney-Lazar 2013). Ramcilovic-Suominen and Kotilainen (2020) outline the policies that limit or stabilize shifting cultivation in Laos, including: (i) resettlement from uplands to lowlands, (ii) promotion of sedentary agriculture, (iii) land use planning and allocation, (iv) market expansion and production of cash crops. Some of those interventions, such as land use and allocation policies and incentives for cash crops farming, have been used to reduce shifting cultivation in our case study.

The REDD+ process in Laos officially started in 2008 when Laos became one of the first REDD+ Readiness countries of the Forest Carbon Partnership facility (FCPF). The Government of Laos received its first REDD Readiness Grant of USD 3.6 million in 2015 (Dwyer and Ingalls 2015; Vongvisouk et al. 2016, MAF 2018). The FCPF invited Laos into the Carbon Fund pipeline in March 2016 (Koch 2017). Since 2016, the main focus of REDD+ has been development of a policy and institutional framework. This process was spearheaded by the Ministry of Forest and Agriculture and the Department of Forestry jointly with their international development partners, including the World Bank and German, Finnish and Japanese governments and partner organizations. In June 2018, the FCPF Carbon Fund accepted the Laos Emission Reduction (ER) Program Document (MAF 2018). The ER Program and Laotian national REDD+ strategy proposed the reduction of deforestation in six selected pilot provinces in the north, including Luang Prabang, Sayabouri, Luang Namtha, Bokeo, Oudomxay and Houaphan, and our fieldwork was hosted in Houaphan Province. Donors and international development partners have been active in these provinces for decades (Lestrelin et al. 2012). This is also where ethnic minorities such as Hmong and shifting cultivation are widespread (Baird and Shoemaker 2007; Ramcilovic-Suominen 2019).

The Lao REDD+ process has been supported by the Forest Investment Program (FIP) and the UN-REDD program, the Japanese government, the World Bank and the Finnish Government. Most recently, substantial and long-term support was provided by the German Federal Ministry for Economic Cooperation and Development (BMZ) through its Climate Protection through Avoided Deforestation (CliPAD) project.^4^ The CliPAD project supported the Lao government to develop a national REDD+ strategy and institutional infrastructure at the national level, including the work under the ER Program. In addition to the project’s activities related to REDD+ policy design at the national level, CLiPAD piloted REDD+ implementation at the village level in Houaphan Province. This is done under CliPAD’s Village Forestry (VF) component, using sustainable village forest management as an entry point (Koch et al. 2015; GIZ 2016, 2017). Under this VF project component various technically complex yet politically sensitive activities took place and various binding local-level institutions were created (Appendix S3).

## Environmental justice and social safeguards in REDD+: Theoretical perspective and analytical approach

With origins in environmental social sciences, a three-dimensional framework of justice has emerged as an important heuristic for unpacking complex and highly sensitive notions of justice with relation to environmental issues (Schlosberg 2004, 2007; Walker 2012; Martin et al. 2013). This framework proposes ‘procedural justice’, ‘distributional justice’, and ‘justice as recognition’ (’recognitional justice’) as three key components of justice. *Procedural justice* concerns the process of decision-making: who participates (what actors and societal groups) and who is represented in the decision-making process (Fraser 2009; Sikor 2013). Representation extends beyond nominal participation to highlight who de facto influences decisions, and whether and how the participating actors represent the entire constituency or concerned group/s (Fraser 2009). *Distributional justice* relates to how the benefits and opportunities and harms and risks, which may result from policy or project interventions, are distributed among different actors and societal groups (Walker 2012). This includes not only benefits and burdens of economic nature, but also those related to rights and responsibilities (Sikor 2013). *Justice as recognition, or recognitional justice* raises the importance of recognizing people’s identities, histories and cultural self-determination (Schlosberg 2004). It is defined as non-discrimination and mistreatment based on people’s backgrounds, cultural and institutional prejudices, bias, systematic discrimination, oppression, stereotyping and stigmatization against certain societal groups (Schlosberg 2007; Fraser 2009). Scholars have argued that, when defined like this, recognition is limited to only cultural aspects and cultural self-determination while bypassing political aspects and power relations (Temper 2018, 2019; Rodriguez 2020). Our findings, as we discuss later, highlight the importance of power asymmetries, the right to political self-determination, and recognition of customary authorities and their governing capacities in shaping justice outcomes.

We apply the three-dimensional environmental justice framework for its significant similarities with the Cancun safeguards dealing with social and environmental justice (UNFCCC 2011). Each of the three justice dimensions relate to one of the social safeuards we outlined in the introduction, namely (i) respect for the knowledge and rights of Indigenous Peoples and local communities, (ii) full and effective participation of Indigenous Peoples and local communities and (iii) enhancement of environmental and social benefits and local livelihoods. Concerning the Free, Prior, and Informed Consent (FPIC) of affected communities prior to undertaking the REDD+ actions, although the Cancun safeguards do not explicitly require it, FPIC is implied through reference to the United Nations Declaration on the Rights of Indigenous Peoples (UNDRIP) (Carodenuto and Fobissie 2015). Our analytical framework (Table 1) draws on the presented theory and the key REDD+ elements that relate to it, including participation, land and forest ownership, carbon benefits, REDD+ payments, and access and rights to resources. We combine these to form four analytical elements (Table 1) covered in the results section. As findings concerning recognition frequently emerged in the context of procedure or distribution, we present recognition jointly with procedure and distribution. In discussion we then further elaborate how recognition and political dimensions, such as power relations and political self-determination shape justice outcomes, arguing for a need to incorporate such elements in theoretical and analytical inquiries on environmental justice.

**Table 1:** Analytical framework

	Participation and recognition	Distribution and recognition
National/Policy design level	1. Participation of different actors and societal groups in REDD+ policy design at the national level	3. Land and forest ownership and carbon rights as policy issues at the national level and implications to different actors and societal groups
Village/Piloted implementation level	2. Participation of different actors and societal groups in REDD+ piloting activities at the village level	4. Distribution of burdens and opportunities, including REDD+ monetary benefits, rights and access to resources, across different actors and societal groups

## Research methods and case study

At the national level, we followed the design of REDD+ policy and institutional frameworks. At the subnational level, we studied a REDD+ pilot project (CliPAD) as it was implemented in two case study villages located in Huaphan Province, in the north of Laos. We refer to the two villages as Ban Lao-Khmu and Ban Hmong, where ‘Ban’ means village and the rest of the village name is derived from the dominant ethnic background of their inhabitants, namely Lao, Khmu or Hmong.

The first author collected in-country primary data in 2017. At the national level, 33 semi-structured interviews were conducted (19 in English and 14 in Lao with the support of a local research assistant; see Appendix S1). These interviews dealt with REDD+ policy design, lasted between 1 and 1.5 h, and with one exception were all recorded, transcribed and translated into English. The piloting of REDD+ implementation was studied through fieldwork in Sam Neua District of Houaphan Province, one of the two Districts where the CliPAD REDD+ pilot project activities were piloted. At the provincial and district levels, 14 structured open-ended interviews were conducted in local languages (duration between 45 and 75 min), and 12 of these interviews were audio recorded, transcribed and translated into English. At the provincial level informants were selected based on their prior involvement in the REDD+ process and CliPAD project activities (see Appendix S3), such as with the provincial REDD+ Task Force, provincial and district REDD+ Action Plan Units, and forest inspection units. At the village level, 31 open-ended interviews were carried out following a structured open-ended interview guide but with a possibility for informants to talk about any other issues of relevance. Two villages were selected based on practical reasons as assessed by the project staff who helped the research team enter the villages. The interviews were carried out in the local languages. Respondents included representatives of Village Authorities, members of Village Land Use and Forest Management Committees and Village Development Funds, and ‘ordinary’ villagers (i.e., villagers who were not affiliated to any of these village institutions) (see Appendix S3). In Ban Lao-Khmu, respondents were generally willing to be recorded (12 interviews were recorded), while in Ban Hmong only eight out of 15 respondents agreed to be recorded. Where interviews were not recorded, we relied on field notes. While interviews allowed the researchers to understand the issues at hand, especially considering the restrictions imposed by the authorities for making longer stays in the villages, constraints in speaking freely was observed among the villagers in both villages, but especially in the Hmong village. This was evident in the significantly lower willingness to be recorded. As a consequence the findings may have hidden details and truths that might have been captured through ethnographic methods such as participant observation or action research.

The villages differed not only in ethnicity but also in terms of village forest size and condition and forest cover and land availability, including land allocated for shifting cultivation, livelihood activities and proximity to provincial towns and markets. This diversity provided within-case diversity. Ban Lao-Khmu was located in proximity to the provincial capital Sam Neua, where road networks facilitated transportation. The village was populated by the dominant *Lao* ethnic group and the relatively well-integrated *Khmu* ethnic minority group, most of whom understand and speak Lao.^5^ The village forest is considered severely degraded and there was a lack of forestland available for shifting cultivation as well as paddy fields for rice cultivation. In this village, many households were resettled, most likely due to a governmental resettlement program, from mountainous areas, which had increased pressure on the existing forest land for shifting cultivation and other forest-based livelihood activities. In addition to farming and non-timber forest products (NTFP) collection, villagers had already adopted a variety of government supported and project promoted income-generating activities, including silk weaving, biofuel crops and livestock rearing. Importantly, many men worked at a brick factory in a neighboring village.

In contrast, the Hmong village was located higher up in the mountains with relatively difficult accessibility. The village was fully populated by a single ethnic minority group—the Hmong. As described in “Politics of exclusion and REDD+ in Laos” section, Hmong people are widely discriminated against by the ethnic *Lao* group which itself, however, includes a number of ethnic subgroups (Evans 2002; Singh 2012). The village forest in Hmong village was in a better condition compared to that of Ban Lao-Khmu and was relatively large in relation to the village population size. This related to the size of the village, its remote location and the rarity of policy and development interventions to which this village was exposed. There was adequate land of about 10 ha/household for shifting cultivation compared to about 1–3 ha/household in Ban Lao-Khmu. Livelihood practices were less diverse compared to Ban Lao-Khmu and there were limited income-generating activities. The villagers engaged mainly in subsistence livelihood activities, including upland rice cultivation, shifting cultivation, livestock rearing, hunting and NTFP collection.

The interview data were analyzed using a directed approach to qualitative content analysis (Hsieh and Shannon 2005). In directed content analysis an existing theory or prior literature is used for seeking or identifying initial cues and concepts in the data, especially in the initial stages of analysis. As analysis progresses, the process become less directed and more flexible, and the researchers can organize and recode the data beyond the concepts and theoretical framework while keeping in mind the empirical context. This means that there is flexibility to allow for new concepts and ideas to emerge from the data. In our case, initial coding was guided by the environmental justice framework, where a first reading of the data resulted in a list of initial code categories influenced by the theoretical concepts. The initial code categories included participation, representation, land ownership, carbon ownership, benefit sharing, rights and access to resources, village forestry, and shifting cultivation. In the process of analysis, the code categories were revised, expanded using new code (sub)categories (e.g., livelihoods, value conflicts, conflicts of interests), or submerged (e.g., livelihoods and shifting cultivation were later submerged with the access to resource code category). Finally, the different codes and subcodes were easily reframed in terms of the broader theoretical concepts related to procedural and distributional justice as presented in Table 1.

## Results: Procedural justice and recognition in REDD+

### Participation in REDD+ policy design at the national level

We first highlight the contradictions between the REDD+ social safeguards agenda and the Lao political and administrative culture that limits public participation of non-governmental actors and that limits, or does not recognize, the cultural and political self-determination of ethnic minorities. This has an important bearing on the CSO and villagers’ participation, representation and recognition, or lack thereof, in the REDD+ processes at the national level. Some of the additional major challenges with regard to participation and recognition originate from, and relate to, the REDD+ design itself, including the tight schedules and the scientific and technical basis of the instrument, which demand highly trained staff not found among the locals.

These limitations are reflected in and explain CSO feeling of waning interest in the REDD+ policy-making process, and we highlight the following key findings with regards to the participation challenge: (1) prioritization of technical soundness over local capacity building; (2) lack of political will to strengthen participation and resolve politically sensitive issues; (3) staged participation as a ‘face-saving’ strategy that led to further participation fatigue in REDD+. The respondents commonly acknowledged that the REDD+ key actors—national level government and international development partners—preferred to work with international organizations (INGOs) and consultants rather than with civil society groups that were smaller and less acquinted with a REDD+. Domestic CSOs generally perceived REDD+ as a foreign affair in which REDD+ funds were mainly used to hire foreigners who do not understand the local political and societal context.

There is a lack of cooperation and responsiveness from the side of INGOs and development partners. They establish their offices here, grab the funds and cooperate with the GoL [Government of Laos]. We do not really enter this field of cooperation. Priority is given to foreigners. The GoL wants the WB [World Bank] to support them that is why they hire international TA [technical assistance] teams to work on REDD+ (#01).

Respondents from the Department of Forestry (DOF) believed that CSOs did not understand the REDD+ process, and that it was also not the best time to involve them since *“the REDD*+ *process is finally going well (…), and perhaps we also do not really involve them. Because we tell them that this is for the GoL [Government of Laos] to decide, because it really is” (#02)*.

In addition to a lack of procedural justice in the process, this statement also reflects a lack of representation and recognition of domestic non-governmental or civil society as an equal ‘stakeholder’ in the process.

A second and related finding concerns how Lao political culture maintains the lack of non-state actor participation and bypasses contentious issues, including ethnicity and land ownership, which we cover in the next section. All respondents thought that CSO participation was not rooted in governmental policy and political culture but was a requirement imposed by international donors. The CSO engagement in REDD+ was understood as part of ‘external’ and safeguards-related donor requirements, even if some respondents from international development partner organizations and international CSOs stated that participation of CSO is not a strict condition for REDD+ funding. In any case, the lack of local CSO involvement in REDD+ also meant that socially and politically contentious issues, including local peoples’ aspiration for political rights and decision-making power, were sidelined.

Interviews highlighted previous failed attempts to involve CSOs and address local people’s concerns and aspirations in forest policy processes, including under the Forest Investment Program (FIP), where a 4.5 million USD fund was allocated for working with local communities, ethnic minorities and CSOs. But when the World Bank held meetings with CSOs without inviting the government, the fund’s activities were abandoned.

The Ministry for Home Affairs got really upset that the World Bank was dealing directly with CSOs, and not going through them, and then surprise, wink-wink, the leadership at the Bank changed. And now there is a new forester at the World Bank who does not want to work with CSOs. They say that it is too politically sensitive. But that is rubbish. It is only that the GoL is not in favor of CSOs meddling with what they consider “their job” [sic] (#03).

It is therefore no surprise that REDD+ project implementers and development partners started to use staged participation as a strategy to legitimize the process while at the same time avoiding contentious issues of rights and empowerment. Staged participation was explained as a process where *some* CSOs are occasionally invited to *some* consultation meetings after which there is no follow-up and participants do not see any results (description deduced from interview #09). This resulted in loss of interest in participating in meetings that had no impacts.

With regards to consideration and consultation of villagers in the REDD+ policy process at the national level, some village consultations were carried out under the Social and Environmental Safeguard Assessment (SESA). The SESA team of four experts were tasked with conducting village consultations in five REDD+ Provinces.^6^ The plan was to cover two districts and four village clusters per province. However, due to time constraints, the consultations were reduced by half. Women and ethnic minority groups were hardly present in these meetings, and where they were, they were not engaged in conversation (deduced from interviews with respondent #03). While those working with the SESA were proud of the process, respondents from civil society and development partners, who were not directly involved in SESA, thought that village consultations were lacking and that the main actors targeted by REDD+ (shifting cultivators) remained unheard: *What is lacking here is still a consultation at the grassroots level, especially with shifting cultivators. If you are saying that one of the key drivers of deforestation is shifting cultivation, they have to be heard. This so far has not happened in Laos (#04).*

### Participation in REDD+ piloting activities at village level

The main platform for involving villagers in REDD+ decision-making at village level was the project meetings. Based on interviews and project documents, two types of village meetings were held in the villages. First, the so-called “non-FPIC meetings” (see also Koch et al. 2015, p. 3), where actors external to the village (from international, national, provincial and district level) invited village authorities and members of the village forest and land committee to jointly plan and discuss the VF management plans, guidelines and project activities. Second, “FPIC meetings” were held to which all villagers were invited. In the “FPIC meetings”, the guidelines, rules and project activities pre-planned in the “non-FPIC meetings” were communicated and discussed with the rest of the villagers. In these “FPIC meetings”, the villagers were consulted on the pre-defined activities, issues and decisions, after which they signed a written consent for those activities and decisions to be carried out.

Asked about the involvement, deliberations and role of villagers in influencing the key decisions, some respondents from district and provincial levels stated: *“After we shared with them the purpose of this plan (referring to the village forest management plan), they gave input to the plan. Whether they like it or not, this is our strategy to manage the forest. The majority of participating villagers understood that” (#05).* This statement explains how and why some Hmong villagers in Ban Hmong chose not to join the meetings because they expected that they would not have much say in the final decision and they feared that their presence would be interpreted as consenting to pre-made decisions. The villagers’ strategy of refraining or withholding is a strategy of revolt and protesting non-recognition of their political agency and self-determination.

In the Ban Hmong village authorities and members of village forest and land committees attended the meetings. Other villagers attended to a lesser degree, with women and youth particularly absent. Women joined if the men were not available. Household obligations and not being sufficiently prepared to speak up in the presence of village and state authorities were given as reasons for women not attending the meetings. This is an aspect that also reveals the lack of recognition of woman by the dominant culture and power structures—both internal or local and external. Another obstacle to attendance and effective participation of the Hmong in meetings was the use of the Lao language, which suggests violence against cultural self-determination. Hmong villagers argued that they were invited to listen to a meeting conducted in a language that most of them did not speak or understand. Finally, the lack of trust in outsiders and their institutional procedures, both the Lao government and foreigners, also played a major role in villagers choosing to limit their participation in meetings and project activities. This distrust led to people fearing that they would lose the forest to the project and to the government. As one respondent said (source from fieldwork notes, #06):

I know how the projects work. We know how Chinese projects work. This is no different. How do we know this is different? We want to protect the forest. But we are afraid someone from the government, together with a Chinese or German company will come and cut our forest and then they will try to sell it back to us. Look at me, look at my house, I cannot afford to pay for the trees.

Unlike in Ban Hmong, meeting attendance was significantly higher in Ban Lao-Khmu, where respondents estimated that 80–90% of village household heads attended most of the FPIC meetings they were invited to. Similarly to Ban Hmong, most of the interview respondents said that the women and Khmu minorities present did not talk much in the meetings. These findings suggest nominal participation and consideration for villagers’ concerns, preferences and opinion in the decision-making process, while focus was placed predominantly on meeting attendance.

While inconsistent responses were gathered concerning the level of participation from provincial and district level officials, the role of mass organizations in the village-level meetings and activities was not contested by any respondents. The Women’s Union, Youth Union and Lao Front for National Construction (LFNC) were all present and actively facilitated the “FPIC” and "non-FPIC meetings”. In contrast, no CSOs were present. The project staff argued that there were no active CSOs in Houaphan Province, and that the most efficient way to organize FPIC in the villages was by involving mass organizations. However, as many CSO respondents commented, there may have been other hidden motives for involving mass organizations rather than the CSOs. Namely, it was known that FPIC village consultations involving CSOs in Sayabouri Province in 2011 contributed to the shutting down of CLiPAD project activities. In this latter case, the FPIC process was short lived because after a year of negotiations the project was relocated outside of Sayabouri Province (Dwyer et al. 2016). As a civil society activist explained:

CliPAD was trying to set up a village consultation process and we started with FPIC in four villages in Sayabouri. But we had made it up to the third village when we were stopped, one telephone call, and CliPAD was shut down in Sayabouri. There were a couple of reasons for this, including that the GoL was excluded from this process and that GIZ and CSOs took too much independence in approaching the villages. The other is that it was a military area we had entered. And third and most importantly the GoL had strong feelings against FPIC as an international principle which they do not accept as valid. Now CliPAD is in Houaphan (Province) and you can tell that FPIC looks very different now (#07).

This situation has implications particularly for recognitional justice, an aspect that we return to in the discussion, because FPIC is a process that most directly aims to promote and ensure self-determination in REDD+. Reluctance to accept FPIC as a legitimate requirement also implies a reluctance to recognize the local socio-cultural and political diversity, including the diversity of informal and traditional governance structures.

## Distributional justice and recognition in REDD+ policy design and implementation

### Land and forest ownership and carbon benefits as policy issues at the national level

The government informants and some respondents from academia and domestic consultants believed that villagers cannot be trusted when it comes to forest tenure and owners and that they are incapable of managing the forests. The shifting cultivators were considered particularly careless and in need of training and education (Ramcilovic-Suominen and Nathan 2020). External development partners and CSOs, on the other hand, pointed to a lack of political will to grant more rights and roles to villagers as being an obstacle to community or village forest ownership.

Respondents from the national level, especially those working for governmental offices, believed that the prospects for villages to legally own and manage land and forests were low. As a respondent from academia put it: “*No project in Laos will be able to shift the drift toward community-owned village forests, not in Laos, not any time soon” (#08).* Many governmental officials argued that community forestry and associated forest and land rights and ownership were not a good idea in Laos, given the ‘lack of capacities’ and high poverty levels. Some argued that there was a possibility that villagers would sell the lands and forests to foreign investors if they had the legal land ownership (deduced from interviews #02, #08). These arguments underline non-recognition of the existing customary or informal structures and authorities by their formal counterparts, even if in many places such customary institutions are well preserved and locally respected across Laos (UNDP 2011).

Civil society, both domestic and international, on the contrary believed that communities should be recognized as legal owners of village forests—and of carbon too—when and if carbon titles are distributed for those forests. They thought that the lack of political will to recognize local communities as guardians and managers of the forest (rather than a shortage of community “capacities”) created a bottleneck in their efforts to promote village forest and land ownership beyond REDD+. This lack of political will was linked with lucrative land and agricultural investments. Respondents stressed that as long as the government maintained control over land and forest, local people could be evicted from the land whenever an investment opportunity arose.

As far as carbon ownership is concerned, it is worth noting that many respondents saw this concept as abstract and unclear. Those who were tasked with developing the carbon ownership policy options explained that to be functional, REDD+ does not need to define carbon rights and ownership but only the basis for sharing the monetary benefits. This approach not only contradicts the REDD+ donor requirement of clear carbon rights but it also decouples benefit streams from rights and ownership, which are defined and embedded within the power relations and self-determination aspects. Decoupling benefits from rights allows REDD+ actors to continue the process without disturbing the dominant power structures and relations. This comes at the expense of local people’s rights and benefits and with the added cost of insufficient transparency regarding how benefits are shared.

Many development partners said that the Lao government does not pay much attention to these aspects and assumes that since they own the forest, they also own the carbon in the forest, and it would be the government that will manage and distribute REDD+ monetary benefits. The benefits sharing working group, consisting of domestic and international consultants, were at that time of interviews deciding the basis for sharing benefits, including which agency would manage REDD+ funds and which mechanism would be used to distribute the funds. The working group used the benefit sharing options proposed by CIFOR (Pham et al. 2013) as a starting point. Several interviewed respondents suggested that the most appropriate option would be to set up a scheme similar to that for timber benefits sharing under the DOF. As one respondent explained: *“The way it works with timber is that the GoL owns the timber, the GoL sells the timber and then the communities get a percentage of those benefits. So the GoL owns carbon, the GoL sells carbon, it goes the same way”* (#04).

The REDD+ benefit sharing group discussed the option of installing a REDD+ fund under the existing forestry fund managed by the Department of Forestry. A Carbon Benefit Sharing Decree similar to Timber Revenue Benefit Sharing Decree would be enacted that would define distribution of REDD+ payments. The policy makers envisaged that the benefits should be in the form of support for village development projects rather than financial benefits, because the REDD+ payments were likely to be minimal. They foresaw loans with low interest rates that would be used to channel REDD+ money to villagers. To access loans, villages would need to write a proposal about how they intended to spend the REDD+ money they applied for, and the proposals would need to be approved by a Board. This idea reflects the approach of the Village Livelihood Development Grants commonly implemented across the country (Ramcilovic-Suominen and Kotilainen 2020). However, this approach appears inconsistent with the REDD+ framing of distributional justice as the enhancement of social benefits because it requires villagers to apply for funds that are generated from their sacrifices in conserving forest.

As an example of how this national appropriation of local benefits works in practice, we refer to an important development that further triggered resistance among villagers in Ban Hmong. During the land use planning process for the REDD+ project, an area previously used for shifting cultivation was allocated as a forest protection area. But the villagers in Ban Hmong would not receive compensation for the lost access to this village forest areas nor further explanations except that this is how it was marked in the existing forest maps. Instead, they would need to propose a development project and compete with other villages in the region for the REDD+ funding.

### REDD+ monetary benefits and access and rights to resources in the REDD+ pilot villages

The possibility of generating and distributing performance-based payments under REDD+ was not revealed to the villagers. The project staff justified this as an intentional choice to avoid raising expectations. Respondents from international development agencies argued that since the expected REDD+ performance-based payments were likely to be insufficient to be shared in cash, this funding could instead be used to advance some of the project activities, such as implementation of village management plans, land titling etc. In this way, project implementers proposed strategies that ensured continuity of their own work, which could potentially benefit villagers:

It makes no sense at the end to pay one dollar to villagers per year. It makes no sense. Depending on how much money they could get, they could for example select villages that already have developed village forest management plans, and then use some of the REDD+ money to implement the village plan, or something like that. And that could work. The other option would be to take this money and to work on land use planning and titling, because that is expensive, so REDD+ money could support that process (#10).

Concerning access to forest-based livelihood activities, all respondents in both villages explained that the project has limited their rights in terms of shifting cultivation, tree felling for individual household purposes (e.g., house construction), hunting, fishing, and collecting NTFP. The only improvement felt by the majority of villagers in Ban Lao-Khmu was the right to participate in meetings related to village forest affairs. This indicated a positive trend in procedural justice and also political recognition. The same perception was not shared in Ban Hmong, where about a half of the informants thought that this right had not changed, while the other half thought that the right to participate had decreased. They argued that with the arrival of the REDD+ project, they could no longer decide where and when to hunt and fish and had to approach the district authorities for written permits if they wanted to fell trees, which is an activity that previously required the approval only of the village head.

These findings are consistent with CliPAD village forestry guidelines (PLUP 2009; GIZ 2016). For example, articles 3–6 of the participatory land use planning manual (PLUP 2009) outline the various prohibitions and limitations, including hunting, fishing, tree felling and farming. As GIZ (2016, p. 8) notes:

Very often these rules already exist but have not yet been put in writing and have not been implemented in the villages. The village rules take into consideration the traditions of the village in terms of forest management, but they also bring in the new forest categories and updated policy aims, as prescribed by the forest policy and law.

This statement raises an important question of what it means for a law to “exist” if its existence does not go beyond the paper on which it is printed. While forest laws and policies are enacted and updated constantly at the national level, their implementation and therefore importance is often negligible in places where such laws did not have practical implications, until the moment they were brought into practice through various interventions. In this case, REDD+ interventions.

## Discussion: Justice struggles in donor and nationally driven REDD+

In the discussion, we first highlight the challenges in implementing REDD+ social safeguards related to the Lao authoritarian regime, donor history of trial and error in promoting imported visions of democratic procedures, and other inherent limitations of REDD+ as a ‘nationally driven’ process. Second, we highlight some of the limitations of the three-dimensional environmental justice framework. Namely, we argue that in addition to culture, there is a need to incorporate elements that explicitly relate to politics, in particular power relations, political self-determination and local people’s capacities and aspirations for self-governance based on traditional and customary structures.

The authoritarian political context significantly hinders democratic engagement of non-governmental actors, including CSOs and local people, in natural resource governance in Laos (Stuart-Fox 2005, 2006; Sims 2017; Creak and Barney 2018; Ramcilovic-Suominen 2019). The current REDD+ donors have been working in Laos for decades (Lestrelin et al. 2012; Broegaard et al. 2017) and in the past they have attempted through their development work to promote some of the ‘best practices’ regarding participatory planning and recognition of customary rights (Fujita and Phanvilay 2008). Yet, paradoxically, these initiatives have often resulted in exclusion of villagers, and in increased poverty, land loss and customary rights insecurity (Ducourtieux et al. 2005; Fujita and Phanvilay 2008; Creak and Barney 2018).

While the current practice obviously challenges the efficacy of social safeguards and just outcomes, this political culture itself and lack of inclusiveness at times it seems to help project managers and donors to implement their projects easily without political debate. Procedural justice in our case study was often reduced to ‘staged participation’, which helped bypass contentious issues such as local people’s rights, ownership and decision-making power. This in turn explained the lack of debates on whether and how local people’s rights relate to benefit streams and to distributional justice more broadly.

The REDD+ techno-scientific approach (Myers et al. 2018) and the donors’ imposition of urgency in handling political issues inherent in REDD+ further discourage debates on locally relevant issues, such as customary rights, local people’s political agency and the social safeguards and justice. Ensuring socially relevant issues are identified, recognized, and safeguarded requires time which was not made available in the REDD+ interventions in Laos. The project working mentality and its associated sense of urgency is one of the reasons for working exclusively with international consultants rather than domestic actors (see also Jagger et al., 2014). The hurried SESA process in the villages, for example, did not allow enough time for communities to reflect on the issues at hand. Both these shortcomings explain the difficulty with which REDD+ is moving ahead in Laos (Vongvisouk et al. 2016) as elsewhere (McDermott and Ituarte-Lima 2016; Lund et al. 2017; Massarella et al. 2018). Scholars report similar challenges in REDD+ processes across Southeast Asia (Nathan and Pasgaard 2017; Satyal et al. 2018; Hoang et al. 2019; Milne, et al. 2019) and in other REDD+ countries (Špiric et al. 2016; Saeed et al. 2018).

As far as the three-dimensional justice framework is concerned, our findings revealed that the lack of political and cultural self-determination as well as power asymmetries between state and non-state actors, lack of local people’s empowerment and failure to recognize customary and traditional structures and rules, all shape justice outcomes. Ethnicity, gender, and historical relations with the state emerge as critical factors shaping the conditions for procedural, distributional and recognitional justice. This is evidenced by the different level of participation, involvement and trust in external actors and their initiatives in the two study villages inhabited by ethnicities with different socio-cultural identities and relations to the state. The fact that Hmong people have a distinct history, culture and the political engagement was largely ignored in the REDD+ pilot project design and implementation, which not only acted as a barrier for Hmong involvement but also contradicted FPIC principles for cultural self-determination. Although REDD+ project meetings were open to all villagers regardless of their ethnicity or gender, ethnic Lao reported the highest acceptance of the project followed by the Khmu in Ban Lao-Khmu, while such acceptance was negligible among the ethnic Hmong in Ban Hmong. Many Hmong people seemingly protested against the project interventions by choosing not to participate in the meetings. Similarly, women and youth of both sexes were the least involved societal groups in both villages, regardless of ethnicity. This reflects the relevance of a local culture where male and household heads dictate decisions about forests.

While the three-dimensional environmental justice framework is a robust and prominent heuristic, it also faces increased critique (Menton et al. 2020) for the following main reasons: (i) an anthropocentric focus and focus on current generations only (Pellow 2018), (ii) the dominance of Western imaginaries of state, society and nature (Alvarez and Coolseat 2018), and (iii) failure to recognize that state and formal institutions in some cases help maintain societal injustices (Pellow 2018). We confirm the relevance of the last two critiques, as we find that state policies, reinforced by ‘nationally driven’ climate action, reproduce societal injustices and aggravate cultural and political recognition, and that donor defined ideas are imposed (e.g., seeing forest though a carbon lens) on top of local ideas regarding forests and nature. The three-dimensional environmental justice framework focuses predominantly on distribution, participation and recognition of other people and their cultural identities *within* the dominant governance structures and initiatives. Yet this framework has a narrow focus on the political conditions and politics of subjects that are on the margins or outside such dominant governing system, even if this greatly alters justice for those subjects (Alvarez and Coolseat 2018; Temper 2019; Rodriguez 2020). Historical and current power relations, local people’s struggles for political self-determination and self-governing through traditional indigenous practices and institutions remain out of scope and therefore hard to capture using this framework (Temper 2019). Such aspects are nonetheless crucial in multi-cultural and multi-ethnic societies and countries with inherent and historically rooted mistrust and conflicts between the state and its citizens, and perhaps especially in authoritarian settings, where the state seeks to consolidate control over less regulated spaces, communities and resources. Using justice frameworks that overlook such political complexities may lead not only to overlooking important justice struggles but may also produce new forms of injustices and struggles (Rodriguez 2020). Incorporating political context, power relations, self-determination and self-governing as an explicit set of justice dimensions is crucial. Emerging justice frameworks, including decolonial and epistemic (Alvarez and Coolseat 2018; Rodriguez 2020) and critical environmental justice (Pellow 2018; Carrillo and Pellow 2021), bring up many of these issues and may pave the way for more holistic and context specific justice frameworks, even if more work is needed to strengthen their analytical potential and applicability.

## Conclusions: Toward more just forest climate policies

In conclusion, we want to draw attention to the conditions that promote justice in forest-based climate policies and in that way draw broader implications and provide policy recommendations based on our case study. First, we highlight the importance of scrutinizing the actors and policy networks involved in policy design and implementation, but also in the definition of the policy problems that deserve policy solutions. Widening the circle of actors to provide room for the voices of less powerful, marginalized, and vulnerable groups in policy-making improves the likelihood that the needs, concerns, identities, and political preferences of Indigenous Peoples, women, and ethnic minorities are recognized. This would strengthen their recognition and self-determination and, in turn, the procedural and distributional justice of these policies and would bring us a step closer to more just climate policy and governance.

Rather than communicating pre-defined policy problems and solutions and then asking local people to participate and commit to a policy initiative, it is crucial to co-define policy problems and co-design policy solutions together with those directly affected by the policy interventions. Doing so requires a significant change in practice in terms of a shift in power relations, cooperation, and recognition of marginalized groups in the domestic and international policy and governance arena. Yet, despite the challenges in the context of forest and climate policies in Laos (Kenney-Lazar 2013; Broegaard et al. 2017; Cole et al. 2017; Mustalahti et al. 2017) as elsewhere (Li 2007; McDermott et al. 2012; Sanders et al. 2017; Saeed et al. 2018; Hoang et al. 2019; Milne et al. 2019), a leap in this direction is a precondition for more just, fairer, and long-term policy and governance initiatives. Meanwhile, there is a fundamental disconnect between REDD+ as a ‘nationally driven’ process and the meaningful implementation of REDD+ safeguards that contradict national legislative frameworks and government priorities.

As our study suggests, the currently dominant actors—national state and international development partners—promote formal rule of law and state policies at the expense of non-state, informal, and traditional rules and practices. For instance, the village forest plans and land use planning processes led to imposition of previously less known formal state policies and rules on top of the existing informal traditional rules and practices. This in turn enabled the state and its disciplinary power to penetrate the spaces and territories inhabited and governed by ethnic minority groups using their customary rules and structures. It is after all clear that actor constellations, structures, and power relations are pre-defined by who is involved, represented, and recognized in policy-making.

Concerning the climate change frontier and how to strengthen justice and fairness in global climate policy and governance, it is important to remember that climate change is entangled with intersectional issues and power dynamics such as gender, race, class, ethnicity, geographic location, and other social and demographic factors (Nightingale et al. 2019). Climate policies that are just and locally tailored must fully consider such intersectional factors, together with socio-cultural and political plurality and complexity. Yet despite calls for focusing on socio-cultural and political dimensions and abandoning techno-managerial approaches in climate change policy solutions (Lövbrand et al. 2015, Nightingale et al*.* 2019), much remains to be done.

As far as justice in REDD+ is concerned, carbon payments represent the main incentive for participating in REDD+ and for respecting social safeguards in the process. Such payments are, however, detached from the local meanings of justice in most of the REDD+ countries and particularly so in countries struggling with social and ethnic conflicts under authoritarian rule. Beyond participation and distribution, justice as recognition, and FPIC as an associated REDD+ safeguard, require recognizing local rights to cultural self-determination in ways that are antithetical to authoritarianism. In addition, we have highlighted that politics, including aspects such as political rights, empowerment, political self-determination, and self-governing authority of indigenous and non-indigenous ethnic minority groups, are crucial preconditions for holistic and durable justice outcomes. The nationally driven nature of REDD+ is highly problematic with regard to enabling such preconditions, especially in authoritarian states. Furthermore, such conditions are unlikely to be addressed by externally defined social safeguards and attaching them to carbon payments. A more just REDD+ process needs to provide space for cultural and political self-determination and for recognition of local customary governing structures, including those of ethnic minority groups, as it is those aspects that nurture procedure, distribution, and justice as a more holistic and locally defined condition. Such an approach might enable safeguarding against violence and injustice being inflicted on culturally and politically marginalized groups by the state or any another domineering actor or societal group. The international community could assist these locally driven and just REDD+ approaches by adjusting the REDD+ focus away from costly and technically complex procedures for carbon measurements and reference levels. Instead the focus must be on supporting locally driven strategies for identifying and addressing drivers of both, forest loss and social conflicts and injustices. Such a refocus also implies a shift in international REDD+ financing away from carbon monitoring and payments and toward supporting local capacities and the locally driven development efforts required for positive change in each country in question.

## Supplementary Information

Below is the link to the electronic supplementary material.

Supplementary file1 (PDF 727 kb)

## Footnotes

1 UNFCC REDD + Web Platform. Fact Sheet of Safeguards: https://redd.unfccc.int/fact-sheets/safeguards.html.

2 For instance, scholars have studied REDD + in terms of stakeholder participation (Boutthavong et al. 2017; Mustalahti et al. 2017), land and property formalization (Dwyer and Ingalls 2015), or role of donor agencies and capacity building (Vongvisouk et al., 2016; Broegaard et al. 2017).

3 See Ramcilovic-Suominen (2019) for a shorter historical overview, and Evans (2002) and Stuart-Fox (1997) for a more in-depth history of the country.

4 The German Government funds CliPAD through the KfW development bank, while the German Development Agency (GIZ) coordinates and implements the project in cooperation with the Government of Laos (http://clipad-laos.org/). CliPAD was launched in 2009 and formally ended in September 2018.

5 Laotian languages consist of four main ethno-linguistic families—Lao-Tai (62.4%), Mon-Khmer (23.7%), Hmong-Iu Mien (9.7%), and Chine-Tibetan (2.9%)—which are officially divided into 50 ethnic groups. The 50 ethnic groups in the country can be further broken down into more than 200 ethnic subgroups. Source: https://laos.opendevelopmentmekong.net/topics/ethnic-minorities-and-indigenous-people/

6 Provinces where SESA was conducted differ from the ER Program provinces and include Borikhamxay, Khammouane, Savanakhet, Attepau and Chmpasak. SESA consultations were carried out in five provinces, six districts and eight village clusters.
