# Supplementary material for: Environmental justice and REDD+ safeguards in Laos: Lessons from an authoritarian political regime
Source: Ambio. 2021 Sep 14;50(12):2256–71. doi: 10.1007/s13280-021-01618-7 (PMC8563914; doi:10.1007/s13280-021-01618-7)
Supplement: Supplementary file 1 — Supplementary file1 (PDF 727 kb) [file 13280_2021_1618_MOESM1_ESM.pdf]

Ambio

Electronic Supplementary Material

This supplementary material has not been peer reviewed

Title: **Environmental Justice and REDD+ Safeguards in Laos: Lessons from an Authoritarian Political Regime**

Sabaheta Ramcilovic-Suominen, Sophia Carodenuto, Constance McDermott, Juha Hiedanpää

APPENDIX S1.

Number of interviews per administrative level

**Organizational category**	**National level**	**Provincial level**	**District level**	**Village level**
Lao state organizations and technical assistance to government	6			
International development partners and technical assistance	8			
Lao private sector	2			
International private sector	3			
Lao civil society organization (CSOs)	6			
International CSOs	4			
Academia and research	4			
State organizations (Agriculture and forestry sector)		3	4	
State organizations (Natural resource and environment sector)		3	2	
State organizations (Forest inspection sector)		1		
State organizations (Planning and investment sector)			1	
Village Head and other authorities or members of forest committees/funds in Ban Lao-Khmu				7
Ordinary villagers in Ban Lao-Khmu				9
Village authorities (Head or elders) and members of forest committees/funds in Ban Hmong				6
Ordinary villagers in Ban Hmong				9
Total	33	7	7	31

APPENDIX S2

Descriptors of interviews used in this paper

**Reference**	**Level**	**Actor Type**
01	National	Domestic CSO
02	National	Government
03	National	International consultant to government
04	National	International development partner
05	District	Government

06	Village	Villager/Ban Hmong
07	National	International CSO
08	National	Academia – academia
09	National	Domestic CSO
10	National	International development partner

APPENDIX S3

CliPAD project Village Forestry component involves the following activities, proceses and institution building exercises

1. *Participatory Land Use Planning (PLUP)* process*,* which delineates village land into forest protection, conservation, and production or village use forest areas.
2. *Village Forest Management Plan,* which lists forest conservation and management activities to be carried out by the villagers, such as pruning, patrolling, fire-break constructions, etc.
3. *Village Land Use and Forest Management Committee* composed of village authorities (i.e. the village heads and elders) which is responsible for implementing the Management Plan.
4. *Village Development Fund* is set to pay salaries for forest management activities, and provide for non-forest dependent livelihood activities, such as livestock raising, weaving, and handicraft.
5. *Village Fund Committee* to administer the village development fund.
6. *Village Forest Management Agreement,* which provides the legal obligation for villagers to adhere to the Management Plan and Regulations, as well as the legal basis for villagers to receive payments for carrying out forest management and forest conservation practices.
7. *Village Regulations* to control all forest-based livelihood activities, such as hunting, fishing, cultivating crops, collecting Non-Timber Forest Products (NTFP), logging, etc.
8. *CliPAD's FPIC* process, where villagers are invited for meetings to ensure their consent to the project, including the Village Forest Management Plan and Village Forest Management Agreement.
